# Implementation of a Rapid, Protocol-based TIA Management Pathway

**DOI:** 10.5811/westjem.2017.9.35341

**Published:** 2018-02-08

**Authors:** Susann J. Jarhult, Melissa L. Howell, Isabelle Barnaure-Nachbar, Yuchiao Chang, Benjamin A. White, Mary Amatangelo, David F. Brown, Aneesh B. Singhal, Lee H. Schwamm, Scott B. Silverman, Joshua N. Goldstein

**Affiliations:** * Massachusetts General Hospital, Department of Emergency Medicine, Boston, Massachusetts; † Massachusetts General Hospital, Department of Neurology, Boston, Massachusetts; ‡ Uppsala University, Department of Medical Sciences, Uppsala, Sweden; § Massachusetts General Hospital, Department of Medicine, Boston, Massachusetts; ¶ Brigham and Women’s Hospital, Department of Neurology, Boston, Massachusetts; || Geneva University Hospitals, Department of Radiology, Geneva, Switzerland

## Abstract

**Introduction:**

Our goal was to assess whether use of a standardized clinical protocol improves efficiency for patients who present to the emergency department (ED) with symptoms of transient ischemic attack (TIA).

**Methods:**

We performed a structured, retrospective, cohort study at a large, urban, tertiary care academic center. In July 2012 this hospital implemented a standardized protocol for patients with suspected TIA. The protocol selected high-risk patients for admission and low/intermediate-risk patients to an ED observation unit for workup. Recommended workup included brain imaging, vascular imaging, cardiac monitoring, and observation. Patients were included if clinical providers determined the need for workup for TIA. We included consecutive patients presenting during a six-month period prior to protocol implementation, and those presenting between 6–12 months after implementation. Outcomes included ED length of stay (LOS), hospital LOS, use of neuroimaging, and 90-day risk of stroke or TIA.

**Results:**

From 01/2012 to 06/2012, 130 patients were evaluated for TIA symptoms in the ED, and from 01/2013 to 06/2013, 150 patients. The final diagnosis was TIA or stroke in 45% before vs. 41% after (p=0.18). Following the intervention, the inpatient admission rate decreased from 62% to 24% (p<0.001), median ED LOS decreased by 1.2 hours (5.7 to 4.9 hours, p=0.027), and median total hospital LOS from 29.4 hours to 23.1 hours (p=0.019). The proportion of patients receiving head computed tomography (CT) went from 68% to 58% (p=0.087); brain magnetic resonance (MR) imaging from 83% to 88%, (p=0.44) neck CT angiography from 32% to 22% (p=0.039); and neck MR angiography from 61% to 72% (p=0.046). Ninety-day stroke or recurrent TIA among those with final diagnosis of TIA was 3% for both periods.

**Conclusion:**

Implementation of a TIA protocol significantly reduced ED LOS and total hospital LOS.

## INTRODUCTION

Transient ischemic attack (TIA) affects 0.3% of the United States population annually and is associated with high risk for stroke or cerebrovascular accident. 1–3 The risk of subsequent ischemic stroke is up to 5% in the first 48 hours, and up to 12% within the first 30 days. 4–7

Data has shown that urgent workup and treatment can minimize this risk. 5, 6, 8–10 Therefore, the early stages of TIA represent a tremendous opportunity for stroke prevention. Many TIA patients present to, or are referred to, the emergency department (ED), and for many healthcare systems the ED represents both the point of first healthcare contact and location for initial workup. However, it is unclear if the emergent workup is best performed in the ED, inpatient unit, or on an outpatient basis. 11–13 Some healthcare systems have developed rapid TIA outpatient clinics, 14–17 but these are not widely available in the U.S. As a result, many patients in U.S. systems receive inpatient hospitalization, and recent study results point to a significant increase in admit rates for TIA. 18, 19

In our hospital we noted substantial variability in workup, both in types of testing and in ED and in-patient length of stay (LOS). In addition, it appeared that there were opportunities to streamline care and improve our ability to risk-stratify patients. Therefore, to optimize quality and efficiency, in 2012 we developed a protocol-based pathway for acute TIA management (Figure). This pathway was based upon existing guidelines including those from the American Heart Association 20 (AHA) (published in 2009). These highlighted the value of clinical information in risk stratification; brain imaging (including magnetic resonance imaging), and cerebrovascular imaging. In particular, they included recommendations regarding the use of the ABCD2 score for risk stratification; therefore, these were included in our pathway.

Finally, other studies had suggested that an ED observation unit (EDOU) may provide an optimized pathway for TIA evaluation, and so we incorporated its use for selected patients. 17, 21, 22 While less common in other countries, EDOUs are increasingly used in the U.S. for patients who require more than a brief ED stay but less than 24 hours of observation or urgent diagnostics. 23 Our EDOU was managed by a nurse practitioner (NP) who was empowered to guide patients who would likely require more than 24 hours for their workup, based on availability of hospital resources at the time. In this analysis, we evaluated whether we could use this pathway to provide consistent streamlined care with shorter LOS without increasing 90-day stroke risk.

## METHODS

### Study Design

We performed a retrospective cohort analysis of patients presenting during the six-month time period before protocol implementation (see Figure for protocol), and then the same six-month time period the following year (six months after protocol implementation). The research protocol was approved by the institutional review board (IRB).

**Population Health Research Capsule**

What do we already know about this issue?

Patients with transient ischemic attack (TIA) are often admitted. Some studies have suggested that ED observation units can provide appropriate care more efficiently.

What was the research question?

What would be the impact on patients’ length of stay and outcomes if we were to implement a TIA protocol incorporating an ED observation unit?

What was the major finding of the study?

Our protocol provided the same high-quality care with reduced length of stay, and no increased risk.

How does this improve population health?

TIA protocols using ED observation units can provide safe and efficient care, returning patients home more quickly, and freeing hospital capacity for patients with greater inpatient needs.

### Study Setting

This study took place at a large, urban, tertiary, academic hospital with approximately 1,000 inpatient beds and approximately 100,000 ED visits per year.

### Population

Patients with suspected TIA were eligible if they presented to our ED (either primarily or in transfer) and if clinical providers determined that a TIA workup was necessary. To capture all eligible patients, we used a number of overlapping methods. First, we queried a hospital-based centralized electronic data registry using the following *ICD-9* codes between January 1, 2011, and December 31,2013: Intracranial Hemorrhage (ICH): 430–432.9; Acute Ischemic Stroke 434.91; and Transient Ischemic Attack (TIA) 435.9. We included the diagnosis of ICH to capture the possibility that some patients may have presented with TIA symptoms but were ultimately diagnosed with ICH. Second, we queried our hospital’s ongoing AHA Get-With-The-Guidelines® data collection, a prospectively collected cohort of all patients with stroke or TIA. 24 Third, we queried our ED electronic record for all patients with chief complaint or final diagnosis that included the term “TIA.” Fourth, we queried our ED electronic record for all patients receiving neurology consultation. Most of the patients captured by this criterion did not actually present with TIA (as they included all neurology consultations), but this method provided a wide net (maximal sensitivity) for all those presenting with TIA symptoms, even if the workup ultimately yielded an alternate diagnosis (such as brain tumor or intracerebral hemorrhage).

A physician then reviewed each medical record to determine whether patients truly had TIA symptoms and whether symptoms had resolved at the time of evaluation. We included patients if clinical providers evaluated and worked up the patient for TIA. For patients with multiple visits, we collected data on the first visit only, and recorded the following visits as adverse events if within the given timeframe of data collection.

### Study Protocol

We performed a structured chart review, collecting data on patient demographics, imaging, workup, ED LOS, and hospital LOS. Two physicians abstracted data. Demographics collected included age, sex, presenting features, and past medical history. We calculated the primary outcome LOS, based on times of registration and transfer – all abstracted electronically; therefore, no inter-rater agreement was calculated. Imaging data included all brain and vascular imaging. Data were collected on workup including any echocardiography or Holter monitoring. We captured final diagnoses of the clinical providers (after the ED workup). Neuroimaging was reviewed for clinically relevant findings (such as ischemic stroke, carotid artery stenosis, tumor, etc). In addition, the physician reviewer independently determined a likely final diagnosis. To evaluate outcome and adverse events, we reviewed the electronic record for followup outpatient visits and inpatient visits, up to 90 days after initial presentation. This electronic record review included data from our hospital as well as six other local hospitals covered by the same IRB.

### Data Analysis

We performed statistical analysis using SAS version 9.4 (SAS Institute, Cary NC). Continuous variables were summarized using median with inter-quartiles and compared using Wilcoxon rank-sum tests. We summarized categorical data using frequency and percentage and compared them using Fisher’s exact tests. A two-sided p-value 0.05 or less was considered statistically significant.

## RESULTS

Our search strategy yielded 3,388 visits, of which 989 (29%) were overlapping. After removing these and repeat visits, 2,399 unique visits remained, of which 1,043 occurred during the time period of analysis. After chart review, 280 patients were found to have presented with transient neurologic symptoms that received evaluation for TIA; 130 patients were worked up for TIA before and 150 after protocol implementation. Table 1 shows the demographics of these two cohorts.

To determine whether our intervention was associated with any changes in processes of care, we examined admission patterns, workup, and outcomes (Table 2). We found that patients admitted to ED observation increased from 27% to 72% (p<0.001). This was associated with a decrease in inpatient admissions from 62% to 24% (p<0.001). Median total hospital LOS (including time in ED, observation, and inpatient) also decreased from 29.4 (interquartile ration [IQR] 18.1–54.8) hours to 23.1 (IQR 15.9–35.7) hours (p=0.019).

To examine whether there was a change in type of patients chosen for TIA workup (for example, providers increasing sensitivity by including lower probability TIA patients for workup), we examined the frequency with which final diagnosis was in fact TIA. We found that the distribution of final diagnosis was quite similar (p=0.19) with 45% TIA diagnosis before and 41% after. Of those ultimately found not to have a TIA, similar frequencies of alternative diagnoses were found (Table 2).

We noted that the majority of patients, both pre and post intervention, received brain imaging (99%) and vascular imaging (92%). For brain imaging, 52% of patients received both head computed tomography (CT) and brain magnetic resonance imaging (MRI), while only 4% received both CT angiography (CTA) and MR angiography (MRA). As our observation protocol included preferential use of MRI over CT, we examined whether head CT use changed. Use of head CT decreased from 68% to 58% (p=0.11) and from 32% to 22% (p=0.078) for neck CTA. Yield of various modalities (frequency with which a clinically-relevant, positive finding was diagnosed) is shown in Table 3. Significant findings on head CT were typically findings suggestive of recent infarction. Significant findings on CTA and MRA were typically findings of vascular stenosis or occlusions.

To examine whether our intervention, and its associated shorter LOS, led to higher risk of adverse outcomes, we evaluated risk of TIA or stroke within 90 days of presentation. Table 4 shows that short-term stroke and recurrent TIA rates were approximately 3% both pre and post intervention.

## DISCUSSION

Overall we found that implementation of a TIA clinical pathway, incorporating the use of an ED observation unit for selected patients, shortened hospital LOS, and we found no evidence for increased risk of followup stroke.

After this study was completed, the American College of Emergency Physicians (ACEP) published guidelines for TIA management. 25 These guidelines suggested not using the ABCD2 score to determine which patients could be discharged from the ED before a complete workup, instead highlighting the value of urgent imaging. As the American Heart Association suggests, there is substantial value in using a tissue-based definition of TIA rather than time-based, a definition requiring brain imaging to evaluate for signs of areas of infarct. 20 In addition, many authors have found that clinical prediction scores that do not use imaging (such as the ABCD2 scores) do not appear adequately sensitive to safely guide which patients can be discharged prior to urgent evaluation. 26

We note that we did not (either before or after implementation of our clinical pathway) use the ABCD2 or another clinical score to discharge patients prior to urgent workup. Instead, our clinical pathway used this score to stratify which patients could receive their workup in an EDOU rather than as an inpatient. In fact, the ACEP guidelines included many elements that we had already included, such as “when feasible, physicians should obtain MRI with diffusion-weighted imaging (DWI) to identify patients at high short-term risk for stroke;” “When feasible, physicians should obtain cervical vascular imaging to identify patients at high short-term risk for stroke;” and “a rapid ED-based diagnostic protocol may be used to evaluate patients at short-term risk for stroke,” 25 As a result, our clinical pathway, although designed before these guidelines were published, remains concordant with them and remains in place today. It is also concordant with many suggested pathways in the literature. 26

Many centers have studied the optimal location for TIA workup. These have included outpatient TIA clinics where assessment, workup, diagnosis and treatment can be efficiently performed. These may reduce unnecessary or avoidable hospital admissions. 14, 27 Such clinics may be less common in the U.S. than in countries with single-payer healthcare systems. Others have studied the use of EDOUs, typically structured as an ED or inpatient unit but designed for patients who need more than an initial ED workup, but less than 24 hours of observation or evaluation. These can be lower cost (to the healthcare system) than an inpatient admission, and are increasing in popularity in the U.S. 23 Some have found that these EDOUs can minimize inpatient admission for lower risk TIA patients in a safe manner. 19, 22 Our results are consistent with these findings. It appears that urgent workup of low- and intermediate-risk patients can be safely performed in an EDOU, reserving high-acuity, in-hospital beds for just the highest risk patients or those with clinically significant findings on workup. Such efforts successfully reduced not just hospital admissions, but total hospital LOS for all patients, while ensuring all necessary workup was performed in the acute setting.

One common limitation in TIA studies is that they often include only those patients with final diagnosis of TIA. However, many patients present with symptoms concerning for TIA, who are later determined to have an alternate diagnosis. The strength of this study is that we included all patients for whom ED providers suspected TIA. However, as a result only about half of patients finally did have TIA, similar to other findings. 28, 29 One interesting finding was that while use of head CT was reduced, approximately half of patients still received one. Many patients received brain imaging with both CT and MRI, which may expose patients to avoidable radiation risk, as CT did not appear to offer additional information beyond what MRI could provide. It may be that providers wished to obtain head CT to screen for an emergent process before initiating the observation protocol and awaiting MRI (which takes much longer).

## LIMITATIONS

Our study had a number of limitations. First, it was limited to a single center, and our care pathways may not be the same as at other centers. Second, observational studies comparing before/after change implementation run the risk of other changes in clinical care happening at the same time. However, we are not aware of any changes in national or local recommendations for TIA workup during the time frame of this study. Third, adjudication of which patients truly had a TIA can be subject to inter-rater variability, which has been shown to be significant in several other studies. 30, 31 Fourth, as this was a retrospective study, our followup was limited to hospital records at our hospital and six other affiliates. We may have missed that some patients developed adverse events that were managed at other hospitals, or doctor offices. Fifth, we were unable to evaluate changes in cost, as cost data is typically not publicly available at our center.

## CONCLUSION

In conclusion, we found that implementation of an ED observation-based TIA pathway led to shorter length of stay, with no evidence for increased risk of follow-up stroke.

## Figures and Tables

**Figure:**
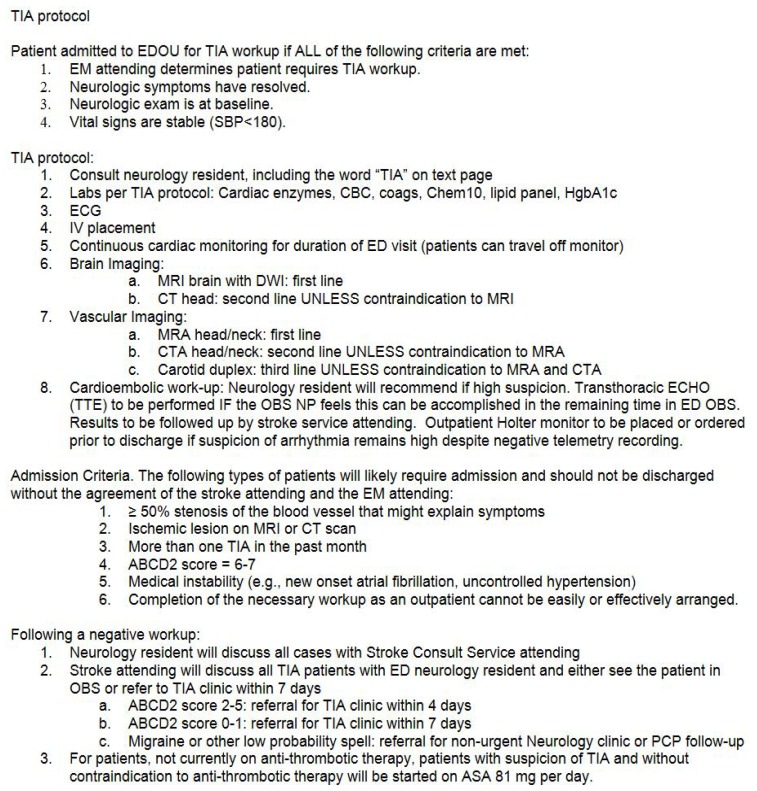

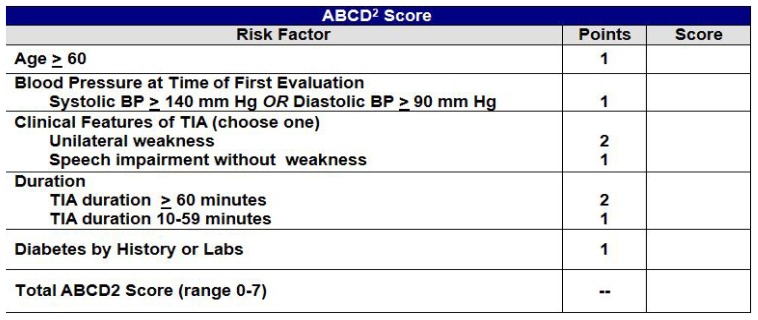
Protocol for transient ischemic attack (TIA).

**Table 1:** Demographics of study population presenting with transient neurologic symptoms.

Variable	Pre-intervention; n= 130	Post-intervention; n= 150	P value
Age, years; median (IQR)	70 (58–79)	68 (52–79)	0.16
Sex, Male (%)	67 (52)	73 (49)	0.72
Diabetes (%)	29 (22)	20 (13)	0.059
Initial SBP, mmHg Median (IQR)	152 (134–172)	150 (129–172)	0.54
Initial DBP, mmHg Median (IQR)	81 (71–90)	80 (70–90)	0.81
ABCD2 score			0.43
0–1 (%)	2	3	
2–5 (%)	72	78	
6–7 (%)	26	19	

**Table 2:** Processes of care.

	Pre-intervention; n= 130	Post-intervention; n= 150	P value
Disposition			<0.001
Discharged home from ED (%)	15 (11)	5 (3)	
Admit ED obs (%)	35 (27)	108 (72)	
Admit inpatient (%)	80 (62)	36 (24)	
Workup			
Head CT (%)	88 (68)	87 (58)	0.087
Neck CTA (%)	41 (32)	33 (22)	0.039
Brain MRI (%)	108 (83)	132 (88)	0.44
Neck MRA (%)	79 (61)	108 (72)	0.046
Carotid US (%)	14 (11)	15 (10)	0.99
Echocardiography (%)	50 (38)	52 (35)	0.36
Holter monitor while admitted	39 (30)	38 (25)	0.015
Holter planned after discharge	9 (7)	20 (13)	0.42
Length of stay			
ED LOS in hours; median (IQR)	5.7 (4.0–7.8)	4.9 (3.5–6.4)	0.027
ED OBS LOS in hours, of those admitted to obs; median (IQR)	10.7 (6.0–17.3) N=46	15.6 (8.8–20.5) N=124	0.034
Inpatient LOS in hours, of those admitted; median (IQR)	39.1 (20.3–84.3) N=84	61.8 (39.4–98.2) N=37	0.057
Total Hospital LOS in hours; median (IQR)	29.4 (18.1–54.8)	23.1 (15.9–35.7)	0.019
Final diagnosis (%)			0.18
TIA	59 (45)	61 (41)	
Stroke	19 (15)	15 (10)	
ICH	0 (0)	0 (0)	
Migraine	6 (5)	4 (3)	
Infection	0 (0)	0 (0)	
Tumor	0 (0)	3 (2)	

*ED*, emergency department; *OBS,* observation unit; *CT*, computed tomography, *CTA*, computed tomography angiography; *MRI*, magnetic resonance imaging; *MRA*, magnetic resonance angiography; *US*, ultrasound; *LOS*, length of stay; *IQR*, interquartile range; *ICH*, intracranial hemorrhage.

**Table 3:** Yield of neuroimaging: For the purposes of this analysis, imaging was operationally defined as ”positive” if there were clinically relevant findings such as ischemic stroke, carotid artery stenosis, or tumor.

Imaging modality	Pre-intervention	Post-intervention	P value
Head CT positive	3/89 (3%)	4/89 (4%)	0.99
Brain MRI positive	33/109 (30%)	29/131 (22%)	0.18
Neck CTA positive	21/40 (53%)	10/33 (30%)	0.063
Neck MRA positive	12/79 (15%)	6/108 (6%)	0.042
Carotid US positive	7/15 (47%)	5/15 (33%)	0.71

*CT*, computed tomography, *CTA*, computed tomography angiography; *MRI*, magnetic resonance imaging; *MRA*, magnetic resonance angiography; *US*, ultrasound.

**Table 4:** Adverse events in those with final diagnosis of transient ischemic attack.

Variable	Pre-intervention; n= 59	Post-intervention; n= 61	P value
Followup at our hospital outpatient neuro clinic (%)	25 (42)	31 (51)	0.37
Recurrent TIA within 90 days (%)	2 (3)	0 (0)	0.24
Stroke within 90 days (%)	0 (0)	2 (3)	0.50
